# Editorial: Metabolic barriers in cancer and cancer therapy

**DOI:** 10.3389/fonc.2024.1411579

**Published:** 2024-05-08

**Authors:** Sascha Kahlfuss, Jérôme Paggetti, Martin Böttcher

**Affiliations:** ^1^ Institute of Molecular and Clinical Immunology, Medical Faculty, Otto-von-Guericke University Magdeburg, Magdeburg, Germany; ^2^ Institute of Medical Microbiology and Hospital Hygiene, Medical Faculty, Otto-von-Guericke University Magdeburg, Magdeburg, Germany; ^3^ Health Campus Immunology, Infectiology and Inflammation (GCI), Medical Faculty, Otto-von-Guericke University Magdeburg, Magdeburg, Germany; ^4^ Center for Health and Medical Prevention (CHaMP), Otto-von-Guericke-University, Magdeburg, Germany; ^5^ Tumor Stroma Interactions, Department of Cancer Research, Luxembourg Institute of Health, Luxembourg, Luxembourg; ^6^ Department of Hematology, Oncology and Cell Therapy, University Hospital Magdeburg, Otto-von-Guericke University Magdeburg, Magdeburg, Germany

**Keywords:** cancer metabolism, immunometabolism, cancer therapy, therapy resistance, personalized medicine

Cancer is a complex and heterogeneous disease with various entities originating from multiple tissues/sites with different genetic backgrounds. However, all types of tumors are characterized by a dysregulated cellular metabolism. This not only fuels tumorigenesis but also confers growth advantages and resistance to immune cells and (immune-based) therapy. Outstanding research efforts over the past 20–30 years have led to the emergence of three major concepts in the field of cancer immunometabolism: 1) metabolic competition, 2) secretion of regulatory (onco-)metabolites, and 3) induction and recruitment of tolerogenic innate and adaptive immune cells by providing a metabolically favorable microenvironment for these cell types. In terms of metabolic competition, tumor cells have often undergone metabolic rewiring that allows them to consume available metabolites, such as glucose, fatty acids, and amino acids, more efficiently and abundantly than their attacking immune cells. This gives them an advantage in growth and proliferation while disarming the immune cells and hindering their ability to mount an effective anti-tumor immune response (1, 2). At the same time, this metabolic rewiring leads to the secretion of large amounts of metabolic by-products, such as lactate, kynurenine, or reactive oxygen species. While malignant cells have evolved mechanisms to cope with this overabundance of metabolites, many immune effector cells, including T and NK cells and tumorigenic macrophages, are detrimentally inhibited in their function (3–5). The altered metabolic microenvironment leads to the accumulation of tolerogenic immune cells, such as Tregs and myeloid-derived suppressor cells (6, 7). The latter cell types are more resistant to the ‘toxic’ metabolites secreted and do not rely on metabolic pathways/substrates that are primarily used and depleted by tumor cells. The metabolic status of malignant cells can create metabolic barriers for immune cells and immune-based therapies at multiple levels. Therefore, understanding the underlying mechanisms is a crucial goal of current research. This will provide more precise targets for therapeutic intervention.

The current Research Topic frames recent developments in this context and summarizes current knowledge.

The articles by Aizaz et al. and Jantz-Naeem et al. focus on the interplay between the tumor microenvironment (TME) and immune checkpoints as metabolic regulatory circuits. Macrophages, particularly tumor-associated macrophages (TAMs), undergo metabolic reprogramming in response to the tumor milieu, promoting tumor growth and immune evasion. These alterations in macrophage metabolism contribute to the immunosuppressive TME. These pathways represent promising targets for cancer therapy. The CD47 protein, recognized as a “don’t eat me” signal on cancer cells, is involved in metabolic crosstalk between tumor cells and macrophages, influencing immune evasion and tumor progression. Targeting CD47 and other metabolism-directed strategies offers new avenues for cancer treatment by disrupting metabolic interactions within the TME and enhancing anti-tumor immune responses. In addition, immune checkpoint molecules such as TIGIT, PD-1, and CTLA-4 exert regulatory effects on immune cell metabolism within the TME. TIGIT, for example, not only modulates T cell exhaustion but also affects cellular metabolism, potentially altering the balance between pro- and anti-tumor immune responses. Understanding the metabolic regulation of immune checkpoints provides insights into novel therapeutic strategies for cancer treatment, particularly those targeting metabolic vulnerabilities in the TME.

In addition, the manuscripts by Mentoor et al. and Drury et al. highlight the special role of fatty acid metabolism as a critical determinant of cancer progression and response to therapy. Breast cancer cells exhibit a pronounced ability to modulate lipid metabolism, a process that is intricately linked to tumor growth and inflammation within the TME, particularly under conditions of diet-induced obesity (DIO). In addition, dysregulated fatty acid synthase (FASN), a key enzyme in *de novo* lipogenesis, represents a promising therapeutic target. Inhibition of FASN triggers metabolic rewiring in cancer cells, leading to compensatory upregulation of the fatty acid transporter CD36. This upregulation promotes tumor growth and survival, underscoring the importance of fatty acid metabolism in breast cancer progression, particularly in obese individuals. Obesity-induced alterations in lipid metabolism also affect the efficacy of chemotherapy, highlighting the importance of understanding the interplay between fatty acid metabolism and treatment outcomes. Modulation of fatty acid metabolism provides an avenue for the development of targeted therapeutic strategies aimed at disrupting the metabolic dependencies of breast cancer cells and enhancing treatment efficacy. By elucidating the intricate mechanisms governing fatty acid metabolism in breast cancer, novel interventions can be developed to overcome therapeutic resistance and improve patient outcomes.

The manuscripts by Escalona et al., Farsani and Verma, and Wetzel et al. focus on the role of specific pathways and their metabolites in the regulation of cancer progression and persistence. On the one hand, the Warburg effect, characterized by enhanced glycolysis even in the presence of oxygen, underscores the metabolic adaptations of cancer cells. Glucose-derived lactate, a hallmark of the Warburg effect, not only fuels tumor growth but also influences immune cell function within the TME. This glucose-lactate-mediated crosstalk between tumor and immune cells poses a challenge to the efficacy of immunotherapy, highlighting the importance of metabolic intervention in improving treatment outcomes. On the other hand, amino acid metabolism, particularly arginine, glutamine, and branched-chain amino acids (BCAAs), plays a pivotal role in supporting cancer cell growth and immune evasion within the TME. Cancer cells exploit metabolic flexibility to outcompete infiltrating immune cells for essential nutrients, thereby promoting tumor progression and immune suppression. Targeting amino acid metabolism represents a promising therapeutic approach to disrupt the metabolic symbiosis between cancer and immune cells, thereby enhancing immune-mediated tumor control.

Finally, the manuscript by Yi et al. provides insight into the utility of metabolomics as a diagnostic tool, specifically in colorectal cancer (CRC). The metabolomic analysis distinguishes CRC patients from healthy individuals and identifies potential biomarkers associated with disease progression. Monitoring changes in serum metabolites after surgery provides valuable insights into treatment response and disease recurrence. Serum metabolomics offers a comprehensive approach to CRC screening and monitoring, improving early detection and personalized treatment strategies.

In summary, understanding the complex interplay between tumor cells, immune cells, and the TME, as well as metabolic alterations in cancer cells is critical for developing effective cancer therapies and improving patient outcomes.

## Author contributions

SK: Writing – review & editing. JP: Writing – review & editing. MB: Conceptualization, Writing – original draft, Writing – review & editing.
